# PKC-theta-mediated signal delivery from the TCR/CD28 surface receptors

**DOI:** 10.3389/fimmu.2012.00273

**Published:** 2012-08-22

**Authors:** Noah Isakov, Amnon Altman

**Affiliations:** ^1^The Shraga Segal Department of Microbiology and Immunology, Faculty of Health Sciences and the Cancer Research Center, Ben-Gurion University of the NegevBeer Sheva, Israel; ^2^Division of Cell Biology, La Jolla Institute for Allergy and ImmunologyLa Jolla, CA, USA

**Keywords:** protein kinase C-theta, PKCθ, CD28, Lck, signal transduction, costimulation

## Abstract

Protein kinase C-theta (PKCθ) is a key enzyme in T lymphocytes, where it plays an important role in signal transduction downstream of the activated T cell antigen receptor (TCR) and the CD28 costimulatory receptor. Interest in PKCθ as a potential drug target has increased following recent findings that PKCθ is essential for harmful inflammatory responses mediated by Th2 (allergies) and Th17 (autoimmunity) cells as well as for graft-versus-host disease (GvHD) and allograft rejection, but is dispensable for beneficial responses such as antiviral immunity and graft-versus-leukemia (GvL) response. TCR/CD28 engagement triggers the translocation of the cytosolic PKCθ to the plasma membrane (PM), where it localizes at the center of the immunological synapse (IS), which forms at the contact site between an antigen-specific T cell and antigen-presenting cells (APC). However, the molecular basis for this unique localization, and whether it is required for its proper function have remained unresolved issues until recently. Our recent study resolved these questions by demonstrating that the unique V3 (hinge) domain of PKCθ and, more specifically, a proline-rich motif within this domain, is essential and sufficient for its localization at the IS, where it is anchored to the cytoplasmic tail of CD28 via an indirect mechanism involving Lck protein tyrosine kinase (PTK) as an intermediate. Importantly, the association of PKCθ with CD28 is essential not only for IS localization, but also for PKCθ-mediated activation of downstream signaling pathways, including the transcription factors NF-κB and NF-AT, which are essential for productive T cell activation. Hence, interference with formation of the PKCθ-Lck-CD28 complex provides a promising basis for the design of novel, clinically useful allosteric PKCθ inhibitors. An additional recent study demonstrated that TCR triggering activates the germinal center kinase (GSK)-like kinase (GLK) and induces its association with the SLP-76 adaptor at the IS, where GLK phosphorylates the activation loop of PKCθ, converting it into an active enzyme. This recent progress, coupled with the need to study the biology of PKCθ in human T cells, is likely to facilitate the development of PKCθ-based therapeutic modalities for T cell-mediated diseases.

## INTRODUCTION

Protein kinase C-theta (PKCθ) is a key regulator of signal transduction in activated T cells that is linked to multiple pathways downstream of the T cell antigen receptor (TCR; Isakov and Altman, 2002). Engagement of the TCR and the resulting formation of diacylglycerol (DAG) are sufficient for promoting PKCθ recruitment to cell membranes (Monks et al., 1997, 1998). However, localization of PKCθ to the immunological synapse (IS) is entirely dependent on the concomitant ligation of the CD28 coreceptor (Huang et al., 2002). Localization of PKCθ at the center of the IS is essential for activation of signaling pathways that promote T cell-dependent immune responses against distinct antigens and pathogens. While the recruitment of PKCθ to the IS of TCR/CD28 engaged T cells has been extensively studied, information on the molecular basis for this highly selective process has been relatively scarce until recently. The present manuscript provides background information on the molecules involved in this process and describes in more detail the studies that clarified a new mechanism by which PKCθ is being recruited to the center of the IS and is essential for the induction of PKCθ-dependent activation signals.

## THE PKC FAMILY

Protein kinase C was discovered by Nishizuka and colleagues, who demonstrated a new kinase that undergoes activation by limited proteolysis (Inoue et al., 1977), or by translocation to the plasma membrane (PM), where it associates with specific cofactors (Takai et al., 1979). The membrane-associated PKC-activating factor turned to be DAG (Kishimoto et al., 1980). DAG, together with inositol 1,4,5-trisphophate (IP_3_), are products of phospholipase C-mediated hydrolysis of the membrane phospholipid, phosphatidylinositol 4,5-bisphosphate (PIP_2_; Berridge and Irvine, 1984; Nishizuka, 1984). These two second messengers transduce signals from a plethora of activated receptors: the hydrophobic DAG remains bound to the cell membrane where, in addition to PKC, it activates effector molecules such as RasGRP, a guanine nucleotide exchange factor (GEF) for Ras (Lorenzo et al., 2000), while the hydrophilic IP_3_ diffuses through the cytosol and binds IP_3_-receptors, which function as ligand-gated Ca^2+^ channels in the endoplasmic reticulum (ER), thereby triggering the release of free Ca^2+^ ions into the cytoplasm (Takai et al., 1979; Khan et al., 1992; Bourguignon et al., 1994). The utilization of phorbol esters, which mimic the activity of DAG, together with Ca^2+^ ionophores, demonstrated that PKC also plays an essential role in the induction of T lymphocyte proliferation (Truneh et al., 1985; Isakov and Altman, 1987) and reactivation of effector cytotoxic T cells (Isakov and Altman, 1985; Isakov et al., 1987).

Protein kinase C enzymes transduce a myriad of signals from a large number of cell surface receptors that are coupled to phospholipase C and phospholipid hydrolysis. They regulate the function of effector molecules by phosphorylating specific serine and threonine residues. The PKC family includes 10 structurally and functionally related isoforms (for more details, see the first review by Pfeifhofer-Obermair et al., 2012), grouped into three subfamilies based on the composition of their regulatory domains and their respective cofactor requirements (Newton, 1995; Mellor and Parker, 1998). The first subfamily includes conventional PKCs (cPKC; α, βI, βII, γ) that are regulated via two DAG-binding C1 domains organized in tandem near the cPKC amino terminus (Hurley et al., 1997; Johnson et al., 2000; Ho et al., 2001) and an adjacent Ca^2+^ and phospholipid-binding C2 domain (Nalefski and Falke, 1996; Johnson et al., 2000). The second group includes novel PKCs (nPKC; δ, ε, η, θ) that are DAG-dependent, but Ca^2+^ and phospholipid independent for their activity. The third group includes atypical PKCs (aPKC: ζ, λ/ι) that are DAG-, Ca^2+^-, and phospholipid-independent. While PKC enzymes are involved in metabolic processes in different cell types, many studies implicate PKC enzymes in signal transduction networks that convert environmental cues into cellular actions (Rosse et al., 2010). Six of the PKC isoforms, including PKCα, δ, ε, η, θ, and ζ are expressed at varying amounts in T cells (Meller et al., 1999). Immunological studies using different genetic models and pharmacological drugs indicated that distinct PKC isoforms are required for different aspects of the activation and effector functions of T cells. The results suggest that distinct PKC isoforms may serve as drug targets for different T cell mediated adaptive immune responses (Baier and Wagner, 2009).

## PROTEIN KINASE C-THETA

Protein kinase C-theta is a Ca^2+^-independent nPKC isoform exhibiting a relatively selective pattern of tissue distribution, with predominant expression in T lymphocytes (Baier et al., 1993; Meller et al., 1999), platelets (Chang et al., 1993; Meller et al., 1998; Cohen et al., 2009), and skeletal muscle (Osada et al., 1992; Chang et al., 1993). It has a unique ability to translocate to the center of the IS of activated T cells (Monks et al., 1997, 1998) where its full activation requires the integration of TCR and CD28 costimulatory signals (Huang et al., 2002; Tseng et al., 2008; Yokosuka et al., 2008). Engagement of the TCR and the CD28 coreceptor initiates a series of PKCθ-dependent signaling events leading to activation of transcription factors, including NF-κB, AP-1, and NF-AT, which are critical for T cell activation, proliferation and differentiation (Baier-Bitterlich et al., 1996; Coudronniere et al., 2000; Dienz et al., 2000; Lin et al., 2000; Sun et al., 2000; Pfeifhofer et al., 2003). Under certain activation conditions, PKCθ can translocate to the nucleus where it directly associates with chromatin and is involved in the regulation of microRNAs and T cell-specific inducible gene expression program (Sutcliffe et al., 2011). The exact mechanism by which the membrane-bound PKCθ delivers signals to the nucleus has not been fully resolved but studies provided information on a number of effector molecules that operate along this pathway in activated T cells. These studies demonstrated that PKCθ-mediated regulation of NF-κB activity involves the multisubunit inhibitor of κB (IκB) kinase (IKK) complex (Coudronniere et al., 2000; Dienz et al., 2000; Khoshnan et al., 2000; Lin et al., 2000; Bauer et al., 2001).

An important upstream effector in the NF-κB signaling pathway is IκBα, which binds NF-κB in the cytoplasm of resting T cells and mask its nuclear localization signal (NLS), thereby preventing NF-κB translocation to the nucleus (Mercurio et al., 1997; Regnier et al., 1997; Jacobs and Harrison, 1998). IKK-mediated phosphorylation of IκBα signals the protein for degradation (Karin, 1999), exposes the NF-κB NLS and promotes NF-κB translocation to the nucleus and the induction of NF-κB-mediated gene transcription. T cells from PKCθ-deficient (*Prkcq*^– / –^) mice fail to respond to TCR stimulation with degradation of IκBα (Sun et al., 2000), supporting the model whereby PKCθ regulates NF-κB activity through its effect on IKK-IκBα. Some of the effector molecules that link PKCθ to IKK have been identified and include the PKCθ substrate protein, caspase activation and recruitment domain (CARD) and membrane-associated guanylate kinase (MAGUK) domain-containing protein-1 (CARMA1). This scaffold protein is primarily expressed in lymphocytes (Bertin et al., 2001; Hara et al., 2003), where it links PKCθ to NF-κB activation in T cells (Ruland et al., 2001, 2003; Ruefli-Brasse et al., 2003; Xue et al., 2003). Phosphorylation of CARMA1 by PKCθ in TCR/CD28-stimulated T cells, promotes CARMA1 association with the B-cell lymphoma/leukemia 10 (Bcl10) and mucosa-associated lymphoid tissue 1 (MALT1) proteins (Matsumoto et al., 2005; Sommer et al., 2005) leading to recruitment of the trimolecular complex to the IS (Gaide et al., 2002; Che et al., 2004; Hara et al., 2004) and activation of the IKK complex (McAllister-Lucas et al., 2001). Furthermore, overexpression of CARMA1, Bcl10, and MALT1 in T cells, followed by TCR/CD28 stimulation, resulted in the formation of a CARMA1-Bcl10-MALT1 trimolecular complex, where all three proteins were required for maximal activation of NF-κB (McAllister-Lucas et al., 2001; Ruland et al., 2001). It should be noted that in some studies (Khoshnan et al., 2000), but not others (Lin et al., 2000), PKCθ was found to directly associate with members of the IKK complex, particularly IKKβ, suggesting the potential existence of an additional linear route from PKCθ to NF-κB. The transcription factor AP-1, similar to NF-κB, is a primary physiological target of PKCθ (Baier-Bitterlich et al., 1996; Li et al., 2004), while regulation of the NF-AT transcription factor requires cooperation between PKCθ and calcineurin, a Ca^2+^-dependent serine/threonine phosphatase (Pfeifhofer et al., 2003). All three PKCθ-regulated transcription factors have corresponding binding sites on the IL-2 gene promoter, and their binding to the IL-2 gene is essential for optimal IL-2 response (Isakov and Altman, 2002).

While PKCθ-mediated regulation of NF-κB activity in TCR/CD28-stimulated T cells has been studied in great detail, PKCθ is also involved in the regulation of additional cellular functions, and physically associates with additional binding partners. Besides CARMA1, PKCθ physically associate with 14-3-3τ (Meller et al., 1996), Cbl (Liu et al., 1999), Fyn (Ron et al., 1999), Lck (Liu et al., 2000), AKT (Bauer et al., 2001), moesin (Pietromonaco et al., 1998), PICOT (Witte et al., 2000), and the HIV nef protein (Smith et al., 1996). Some of these molecules (i.e., Lck) phosphorylate PKCθ and may affect its activity and/or subcellular distribution, while others, which serve as substrates for PKCθ (i.e., Cbl, 14-3-3τ and moesin) may regulate cellular functions, such as cytoskeletal reorganization.

## DIFFERENTIAL REQUIREMENTS FOR PKCθ BY DISTINCT T CELL SUBPOPULATIONS

Initial characterization of PKCθ-deficient T cells suggested the involvement of PKCθ in cellular responses leading to T cell activation, proliferation, and cytokine production (Sun et al., 2000; Pfeifhofer et al., 2003; Anderson et al., 2006). Subsequent *in vitro* and *in vivo* investigations and the analysis of *Prkcq*^– / –^ mice in different disease models demonstrated differential requirements for PKCθ by distinct T cell subpopulations and during the induction of selected types of immune responses. Thus, PKCθ was found to be essential for the induction of Th2-type immune responses to allergens or helminth infection (Marsland et al., 2004; Salek-Ardakani et al., 2004) and the induction of Th17-mediated experimental autoimmune encephalomyelitis (EAE) that serves as a model of multiple sclerosis (Salek-Ardakani et al., 2005; Anderson et al., 2006; Tan et al., 2006; Marsland et al., 2007; Kwon et al., 2012), and other experimental autoimmune diseases (Anderson et al., 2006; Healy et al., 2006; Marsland et al., 2007; Chuang et al., 2011). In contrast, Th1-dependent mouse resistance to *Leishmania major* infection was intact in *Prkcq*^– / –^ mice (Marsland et al., 2004; Ohayon et al., 2007), and PKCθ was dispensable for CTL-mediated protective antiviral responses, most likely reflecting compensation by innate immunity signals (Berg-Brown et al., 2004; Giannoni et al., 2005; Marsland et al., 2005, 2007; Valenzuela et al., 2009). Consistent with the *in vivo* findings, *in vitro* induction of CD4+ T cell polarization by optimal T cell-antigen-presenting cell (APC) coculture conditions, demonstrated a requirement for PKCθ during Th2 and Th17 cell development, and only moderate effect of PKCθ on Th1 cell development (Marsland et al., 2004; Salek-Ardakani et al., 2004, 2005). Additional studies performed in *Prkcq*^– / –^ mice demonstrated the requirement for PKCθ in the induction of graft-versus-host (GvH) and alloreactive T cell-mediated immune responses (Valenzuela et al., 2009). In contrast, PKCθ-deficient T cells retained the ability to induce graft-versus-leukemia (GvL) responses in allogeneic bone marrow (BM) transplanted mice (Valenzuela et al., 2009).

Protein kinase C-theta also contributes to allograft rejection, as shown by Manicassamy et al. (2008) using an adoptive transfer model. In these studies, *Rag*^– / –^ mice reconstituted with *Prkcq*^– / –^ T cells were unable to reject cardiac allografts, in contrast to the acute allograft rejection observed in the wild-type T cell reconstituted *Rag*^– / –^ mice. However, this was due to lack of PKCθ-regulated expression of anti-apoptotic molecules, such as Bcl-x_L_, which led to apoptosis of the effector T cells; transgenic expression of Bcl-x_L_ in *Prkcq*^– / –^ T cells restored their ability to reject the cardiac allografts. The rejection of cardiac allograft by *Prkcq*^– / –^ mice was only slightly delayed (Manicassamy et al., 2008; Gruber et al., 2009), suggesting compensation**by**other PKC isoforms. Indeed, mice lacking both PKCθ and PKCα, demonstrated a significantly delayed rejection of cardiac allografts (Gruber et al., 2009).

The overall positive role of PKCθ in the activation of effector T cells (T_eff_) and the promotion of adaptive immune responses raise questions about the nature of its function in regulatory T cells (T_reg_) that suppress T_eff_ functions. This issue has recently been partially resolved by Zanin-Zhorov et al. (2010) who found that PKCθ mediates negative feedback on T_reg_ functions. Furthermore, activation of T_reg_ resulted in sequestration of PKCθ away from the IS, and inhibition of PKCθ activity (using the C20 compound) increased the suppressive activity of T_reg_ (Zanin-Zhorov et al., 2010, 2011). *In vivo* studies demonstrated that T_reg_ development in the thymus of *Prkcq*^– / –^ mice is impaired leading to reduced numbers of T_reg_ cells in the periphery (Schmidt-Supprian et al., 2004; Zanin-Zhorov et al., 2010, 2011), although activity of these mature PKCθ-deficient T_reg_ cells was intact (Gupta et al., 2008).

## THE IMMUNOLOGICAL SYNAPSE

Adaptive immune responses are dependent on the effective communication between antigen-specific T cells and APCs. At the very early phase of the activation response, T cells interact via their TCR with cognate peptide-MHC complexes on the surface of APCs and both cell types respond by redistributing their receptors/ligands to the contact area that rearranges as a platform for effective signaling (Dustin and Zhu, 2006). The IS, representing the interface between a T cell and an APC, is formed by specific protein microclustering (Yokosuka et al., 2005) and their segregation into one of two separate regions: a central core [central supramolecular activation clusters (cSMAC)], which contains the TCR and costimulatory receptors, and a peripheral region [peripheral supramolecular activation clusters (pSMAC)], which contains adhesion molecules, such as LFA-1 (Dustin, 2009). T cell surface receptor engagement triggers signaling cascades that result in the recruitment of multiple membrane-anchored and cytoplasmic effector molecules, including kinases, adaptor proteins, and cytoskeletal components, to the IS (Dustin et al., 2010). One of the most prominent proteins to be recruited to the IS of antigen-responding T cells is PKCθ, which localizes at the cSMAC (Monks et al., 1997, 1998). Additional high-resolution imaging analysis by TIRF microscopy demonstrated that PKCθ colocalizes with CD28, and demonstrated that the cSMAC is divided into two structurally and functionally distinct compartments: a central TCR^high^ compartment, where signaling is terminated (Vardhana et al., 2010) and TCR-associated signaling complexes are internalized and degraded, and an outer TCR^low^ “ring” where PKCθ and CD28 colocalize (Yokosuka et al., 2008).

## CD28

CD28 is a type 1 transmembrane glycoprotein that is constitutively expressed as a disulfide-linked homodimer on all CD4+ and CD8+ murine T cells and majority of CD4+ and CD8+ human peripheral blood T cells (Gross et al., 1990; Vallejo, 2005). The human CD28 precursor protein is 220 amino acids long (218 in mouse) and the mature protein possesses 202 amino acids (218 in mouse) due to cleavage of an amino-terminal leader sequence (18 and 19 amino acids in the human and mouse CD28, respectively). In addition, CD28 possesses a cytoplasmic tail of 41 amino acids (38 in mouse) that is critical for signal transduction and coreceptor-induced cell stimulation. Physiological activation of CD28 is mediated by one of two natural ligands expressed on the surface of APCs, CD80, and CD86, which directly associate with a conserved motif [MYPPPY (single amino-acid letter code)] in the extracellular region of CD28 (Kariv et al., 1996; Truneh et al., 1996). Engagement of CD28 provides costimulatory signals that complement or synergize with those provided by the TCR, leading to optimal activation of T cells (Thompson et al., 1989; Harding et al., 1992). CD28 engagement increases IL-2 production (Thompson et al., 1989; Jain et al., 1995; Reichert et al., 2001) and IL-2 receptor expression (Shahinian et al., 1993), and provides survival signals by upregulating the anti-apoptotic protein, Bcl-X_L_ (Boise et al., 1993). In addition, CD28 synergizes with the TCR in providing potent signals for activation of c-Jun kinase (JNK), p38 MAP kinase, and IKK pathways (Su et al., 1994; Harhaj and Sun, 1998), and activation of the NF-κB (Michel et al., 2000; Diehn et al., 2002) AP-1 (Rincon and Flavell, 1994) and NF-AT transcription factors (Michel et al., 2000; Diehn et al., 2002).

The positive role of CD28 in T cell activation was demonstrated in CD28-deficient (*Cd28*^– / –^) T cells, in which TCR engagement in the absence of CD28 costimulation resulted in anergy and/or tolerance induction upon rechallenge with the same antigen (Appleman and Boussiotis, 2003). T cell proliferation and Th2-type cytokine secretion were also severely impaired in *Cd28*^– / –^ mice or wild-type mice treated with CD28 antagonists (Green et al., 1994; Lucas et al., 1995; Rulifson et al., 1997; Schweitzer et al., 1997; Gudmundsdottir et al., 1999). Furthermore, lack of CD28-mediated costimulation led to reduced immune responses against infectious pathogens (Shahinian et al., 1993; King et al., 1996; Mittrucker et al., 2001; Compton and Farrell, 2002) and allografts (Salomon and Bluestone, 2001) and impaired GvH disease (Via et al., 1996), contact hypersensitivity (Kondo et al., 1996), and asthma (Krinzman et al., 1996).

T cell receptor engagement in the absence of CD28 costimulation induces an unbalanced signaling response in which TCR-mediated Ca^2+^ influx predominates. This leads to activation of calcineurin which dephosphorylates NF-AT leading to its nuclear translocation and induction of a limited set of anergy-associated genes resulting in T cell anergy (Macian et al., 2004). CD28, in contrast to the TCR, does not induce a Ca^2+^ response (Lyakh et al., 1997). Instead, CD28-coupled costimulatory signals induce the activation of NF-κB and AP-1, and concomitant AP-1 association with NF-AT, conditions that promote IL-2 production and rescue of the T cells from a state of anergy (Macian et al., 2004).

## SIGNALING DOWNSTREAM OF CD28

**FIGURE 1:**
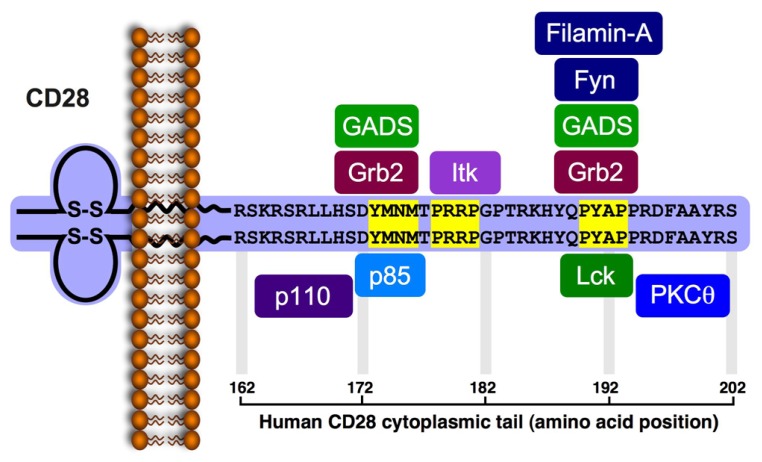
**Signaling motifs in the cytoplasmic tail of the human CD28 and binding partners.** The human *CD28* encodes a 220 amino acid-long protein (218 in the mouse) that includes a leader sequence of 18 residues (19 residues in the mouse). The mature protein (202 residues) possesses a 41 amino acid-long cytoplasmic tail that includes three potential protein-protein interaction motifs (highlighted in yellow). The phospho-Tyr^173^ within the YMNM motif serves as a docking site for the SH2-containg proteins, p85, Grb2 and GADS. The P^178^RRP motif can interact with the SH3 domain of Itk. The P^190^YAP motif can interacts with the SH3 domain of Grb2, GADS and Lck, as well as with filamin-A. Phosphorylation of Tyr^191^ within the PYAP motif creates a docking site for the Lck SH2 domain and enables PKCθ to interact via its V3 domain with the Lck SH3. Studies indicate that Tyr^191^ is important for CD28 and PKCθ localization to the cSMAC, and that the PYAP motif contributes to T cell activation and cytokine expression.

CD28 delivers signals in activated T cells via its cytoplasmic tail, which has no intrinsic catalytic activity, but possesses several protein–protein interaction motifs that enable it to associate with enzymes and other effector molecules (Boise et al., 1993; see **Figure 1**). In resting T cells, non-phosphorylated CD28 associates with the serine/threonine protein phosphatase protein 2A (PP2A), which dissociates from CD28 upon activation induced-phosphorylation of CD28 (Chuang et al., 2000). CD28 triggering by its ligands leads to phosphorylation of tyrosine residues (Raab et al., 1995; Teng et al., 1996; King et al., 1997) in the cytoplasmic tail of CD28, creating new docking sites for different effector molecules that initiate the activation of signaling cascades, and define the costimulatory functions of CD28 (Raab et al., 1995; Andres et al., 2004; Dodson et al., 2009).

The first motif in the human CD28 cytoplasmic tail, juxtaposed to the PM, contains a Y^173^MNM sequence that undergoes tyrosine phosphorylation following the engagement of CD28 and serves as a binding site for the SH2 domain of p85, the regulatory subunit of the lipid kinase, phosphatidylinositol 3-kinase (PI3K; August and Dupont, 1994; Pages et al., 1994; Prasad et al., 1994; Truitt et al., 1994). The methionine residue at the +3 position confers specificity for p85 binding (Takeda et al., 2008), while the asparagine at the +2 position confers additional specificity for the SH2 domain of Grb2 and GADS (Songyang et al., 1993; Raab et al., 1995; Sanchez-Lockhart et al., 2004; Schneider et al., 1995; Harada et al., 2001). The relative concentration of PI3K, Grb2, and GADS at the vicinity of CD28 cytoplasmic tail, and the relative affinity of their SH2 domain for the phospho-Tyr^173^-containing motif likely determine which of the three potential binding partners interacts with the activated CD28 and, hence, the resulting functional outcome. A second, nearby motif possesses the P^178^RRP sequence, and serves as a binding site for the SH3 domain of IL-2-inducible T cell kinase (Itk; Marengere et al., 1997; Garcon et al., 2004). CD28-mediated activation of Itk is dependent on Lck (Gibson et al., 1996), but the actual role of Itk in CD28-induced costimulation is still controversial (Liao et al., 1997; Gibson et al., 1998; Yang and Olive, 1999; Li and Berg, 2005). A third, more distal, P^190^YAP motif serves as a potential docking site for several different effector molecules. These include filamin-A, an actin binding protein and a scaffold for lipid raft formation, which utilizes repeat 10 (amino acids 1158–1246) for interaction with CD28 (Tavano et al., 2006), Grb2 and GADS adaptor proteins, which bind the P^190^YAP motif via their SH3 domain (Okkenhaug and Rottapel, 1998; Ellis et al., 2000), and the Lck and Fyn protein tyrosine kinases (PTKs; Hutchcroft and Bierer, 1994; zur Hausen et al., 1997; Holdorf et al., 1999; Tavano et al., 2004). Both Lck and Fyn were implicated in the early phase of the CD28 signaling pathway (August et al., 1994) and coexpression studies demonstrated that the two PTKs could phosphorylate CD28, primarily on Tyr^173^ at the Y^173^MNM motif, thereby increasing the binding of p85- and Grb2-SH2 to CD28 (Raab et al., 1995). Lck and Fyn were also found to coimmunoprecipitate with CD28 from activated T cells (Hutchcroft and Bierer, 1994), where Lck interacted with the P^190^YAP motif via its SH3 domain (Holdorf et al., 1999; Tavano et al., 2004), and Fyn interacted with the same motif using its SH2 domain (zur Hausen et al., 1997), although other studies indicated no interaction between CD28 and Fyn (Marengere et al., 1997). While presence of the two proline residues in the P^190^YAP motif predicts interaction with SH3-containg proteins, binding studies demonstrated that the Lck-SH3 domain interacts with relatively low affinity (*K*_d_ > 1 μM) with peptides that contain the P^190^YAP motif and correspond to residues 188–202 of human CD28, or 186–196 of murine CD28, respectively (Hofinger and Sticht, 2005).

Other studies demonstrated that Tyr^191^ within the P^190^YAP motif is one of two major phosphorylation sites in CD28-stimulated Jurkat T cells, and the only tyrosine residue within the CD28 cytoplasmic tail that is essential for delivery of costimulatory signals leading to CD69 expression and synthesis and secretion of IL-2 (Sadra et al., 1999). The latter findings raise the possibility that CD28 engagement-induced phosphorylation of Tyr^191^ creates a new and transient binding site for SH2-containing proteins, possibly Lck, since CD28 and Lck were shown to colocalize at the cSMAC (Tavano et al., 2004; Kong et al., 2011). Binding studies provided further support for this hypothesis by showing that a CD28-derived peptide that possesses phospho-Tyr^191^ interacts with the Lck-SH2 domain with a relatively high affinity (*K*_d_ = 2.13 μM; Hofinger and Sticht, 2005), at the range of other SH2-ligand interactions (Bauer et al., 2004). This binding affinity is about three orders of magnitude stronger than that for the Lck-SH3 domain. High affinity binding of Lck-SH2 to P^190^pYAP occurs despite the difference between this sequence and the phospho-YEEI sequence predicted to be the preferred binding site of the Lck-SH2 domain (Songyang et al., 1993). More recent studies indicated that PKCθ can also interact with the cytoplasmic tail of CD28, and that this interaction involves Lck as an intermediate molecule, as discussed below.

## CD28 AND THE IS

Upon binding of its ligand, B7, CD28, similar to the engaged TCR, accumulates at the cSMAC of the IS although the two receptors initiates distinct but complementary signaling pathways. The transient recruitment of CD28 to the immature IS of TCR engaged T cells is very rapid and occurs within seconds of the onset of the calcium signal (Andres et al., 2004). Engagement of the TCR in *Cd28*^– / –^ T cells results in altered, diffuse pattern of distribution of PKCθ and LFA-1 at the IS, suggesting an essential role for CD28 in the initiation and stabilization of the mature IS (Huang et al., 2002; Sanchez-Lockhart et al., 2004). Furthermore, *in vivo* blocking of CD28 impairs the activity of effector molecules, including PKCθ (Jang et al., 2008), and inhibits T cell-dependent immune responses (Linsley and Nadler, 2009). CD28 engagement promotes a cytoskeleton-dependent recruitment of cell surface receptors (Wulfing and Davis, 1998) and signaling molecules-containing lipid rafts that support building the IS and contribute to signal transduction from IS-residing receptors (Dustin and Shaw, 1999; Viola et al., 1999).

More recent studies demonstrated that in activated T cells, CD28 is recruited coordinately with the TCR to form microclusters at the cSMAC (Yokosuka et al., 2008). Upon progression of this initial step, the CD28 and TCR segregate to two spatially distinct subregions within the cSMAC, a central TCR^high^ subregion, where signaling is terminated and TCR-associated signaling complexes are internalized and degraded, and an outer TCR^low^ annular form that contain CD28 clusters, as well as PKCθ. CD28 and PKCθ were physically associated, as shown by PKCθ coimmunoprecipitation with CD28 from a lysate of PMA-stimulated T cells (Yokosuka et al., 2008).

## PKCθ–CD28 INTERACTION AND RECRUITMENT OF PKCθ TO THE IS

T cell receptor engagement polarizes PKCθ and induce its recruitment to the IS, a response that is greatly augmented by CD28 ligation (Huang et al., 2002; Tseng et al., 2008; Yokosuka et al., 2008). Although the recruitment of PKCθ to the center of the IS (cSMAC) of is well documented, information on the molecular basis for this highly selective localization has been relatively scarce. Early studies have shown that PKCθ recruitment to the IS is indirectly dependent on the PI3K interaction motif within the CD28 cytosolic tail (Harada et al., 2001). Thus, mutation of Met^173^ within the mouse YMNM motif, which binds PI3K upon its tyrosine phosphorylation, resulted in decreased ability of CD28 to direct PKCθ recruitment to the cSMAC, and inhibited PKCθ-dependent activation of NF-κB to and the *Il2* gene (Sanchez-Lockhart et al., 2004).

Following the recently reported PKCθ–CD28 association in PMA-stimulated T cells (Yokosuka et al., 2008), we conducted a detailed structure-function analysis of this association in TCR-stimulated T cells (Kong et al., 2011). We demonstrated that PKCθ physically associated with the cytoplasmic tail of CD28 following TCR/CD28 costimulation. Taking advantage of the fact that PKCδ, the closest relative of PKCθ, does not translocate to the IS after T cell-APC interaction (Monks et al., 1997), we compared the amino acid sequence analysis of PKCθ and PKCδ and found that they diverged significantly only in their V3 (hinge) domain, corresponding to amino acids~291–378 of human PKCθ, suggesting a potential role for this region in targeting PKCθ to the IS. Indeed, a V3-deletion mutant of PKCθ (PKCθ-∆V3) or an exchange mutant of PKCθ, in which the native V3 domain was replaced by the PKCδ V3 domain, did not coimmunoprecipitate with CD28, and failed to translocate to the IS (Kong et al., 2011) and to activate PKCθ-dependent reporter genes such as the CD28 response element (RE/AP). Conversely, the isolated V3 domain of PKCθ localized in the center of the IS and associated with CD28. Moreover, T cells recovered from mouse BM chimeras on a *Prkcq*^– / –^ background reconstituted with the same PKCθ mutants failed to proliferate and produce IL-2 in response to CD3/CD28 costimulation, and their ability to upregulate CD69 or CD25 expression was reduced. Given the critical role of the V3 domain in directing the CD28 association and IS localization of PKCθ, we argued that this domain will function as a dominant negative mutant by disrupting the activation-dependent association between endogenous CD28 and PKCθ. As expected, ectopic expression of the isolated PKCθ V3 domain blocked the recruitment of endogenous PKCθ to CD28 and the IS, and severely inhibited PKCθ-dependent functions, including CD25 and CD69 upregulation, T cell proliferation and IL-2 production, and Th2 and Th17 (but not Th1) differentiation and inflammation.

Fine mapping of the PKCθ V3 domain identified an evolutionarily conserved proline-rich (PR) motif (ARPPCLPTP; corresponding to amino acid residues 328–336 of human PKCθ) within the PKCθ-V3 domain, which was required for PKCθ–CD28 association, PKCθ localization to the IS, and induction of PKCθ-mediated functions. Insertion of this motif into the V3 domain of PKCδ enabled this altered PKCδ form to translocate to the IS and activate PKCθ-dependent signal. The two internal proline residues in this motif (Pro-331 and -334) were particularly critical in this regard (Kong et al., 2011).

**FIGURE 2:**
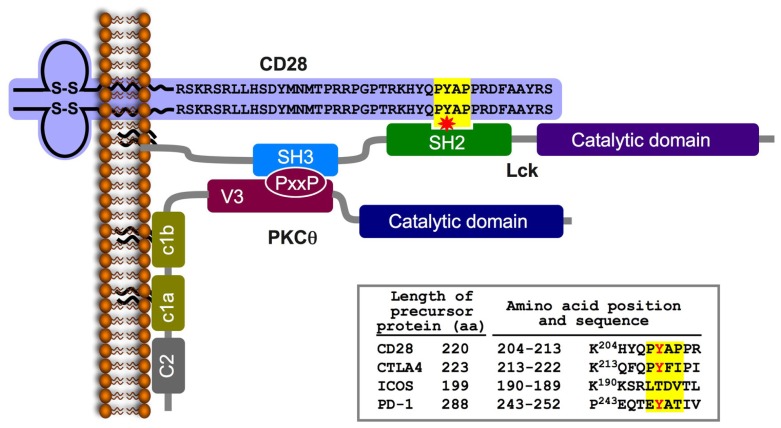
**A schematic model of the CD28-Lck-PKCθ tri-partite complex formed in TCR/CD28-stimulated T cells.** TCR/CD28 engagement triggers the activation of tyrosine kinases and phosphorylation of multiple substrates, including the cytoplasmic tail of the CD28. Phosphorylation of a tyrosine within the PYAP motif (Y^191^ in the mature human CD28) forms a high affinity-binding site for the SH2 domain of the Lck PTK. Lck is an IS-residing molecule in activated T cells; it is tethered to the plasma membrane via its N-terminal palmitic and myristic fatty acids (Paige et al., 1993), and is constitutively associated with the cytoplasmic tail of the IS-residing accessory molecules, CD4 or CD8 (Rudd et al., 1988, 1989; Veillette et al., 1988; Barber et al., 1989; Paige et al., 1993). Simultaneous activation of phospholipase C and hydrolysis of the membrane phospholipid (PIP_2_) forms DAG, which enables PKCθ anchoring to the plasma membrane. Colocalization of PKCθ and CD28 is regulated by an interaction between the PKCθ PXXP motif and the Lck-SH3 domain, which results in the formation of a trimolecular complex comprising CD28-Lck-PKCθ. The inset table shows the amino acid sequence of a region within the cytoplasmic tail of the immature CD28 that includes the PYAP motif (on a yellow background) compared to homologous sequences of three additional members of the CD28 coreceptor family (obtained using the ClustalW multiple sequence alignment program). A partially conserved tyrosine is marked in red.

In trying to more precisely define the nature of the inducible PKCθ-Lck complex, we focused on the potential contribution of Lck kinase. This possibility was considered in view of previous studies demonstrating a functional relationship between CD28, PKCθ, and Lck. First, in stimulated T cells, Lck can be recruited to the tyrosine-phosphorylated distal PR motif (P^190^Y*AP) in the cytoplasmic tail of CD28 via its SH2 and SH3 domains, respectively (Miller et al., 2009; see **Figure 2**), This motif directs the colocalization of PKCθ and CD28 to the cSMAC (Yokosuka et al., 2008) and is apparently involved in additional biological functions, including the stabilization of IL-2 mRNA, reorganization of lipid rafts, and sustained autophosphorylation and activation of Lck at the IS (Holdorf et al., 2002; Sanchez-Lockhart et al., 2004; Dodson et al., 2009). Second, Lck phosphorylates and associates with PKCθ, and mutation of the major Lck phosphorylation site on PKCθ (Tyr^90^) inhibited PKCθ-dependent activation events in stimulated T cells (Liu et al., 2000).

Our further analysis confirmed the physical and functional CD28-Lck-PKCθ link by demonstrating that Lck function as an intermediate to recruit PKCθ to CD28 upon T cell stimulation. The Lck-SH3 domain interacted with the PR motif in the PKCθ V3 domain, while the Lck SH2 domain interacted with phospho-Tyr^191^ in the P^190^YAP motif in the CD28 cytoplasmic tail. Taken together, the above findings demonstrate a unique signaling mode of CD28 and establish the molecular basis for the specialized localization and function of PKCθ in antigen-stimulated T cells.

## THE GLK-PKCθ LINK

Recent studies demonstrated that recruitment of PKCθ to the cSMAC in activated T cells is essential but not sufficient for the full activation of PKCθ and its downstream target molecules. These studies further showed that the germinal center kinase (GSK)-like kinase (GLK) also translocates to the IS of TCR-engaged T cells where it phosphorylates the activation loop of PKCθ, converting it into an active enzyme (Chuang et al., 2011). Of interest, however, despite the importance of PKCθ in the thymic development of natural regulatory T cells (nTregs; Schmidt-Supprian et al., 2004), GLK-deficient mice displayed normal nTreg development (Chuang et al., 2011). These results emphasize the important role of post-transcriptional regulation of PKCθ that occurs at several steps and involve different checkpoints at distinct sites within the activated T cell.

## CONCLUSIONS AND FUTURE PERSPECTIVES

Identification and characterization of the molecular mechanism by which PKCθ associates with CD28 and colocalizes with it at the cSMAC has provided important information relevant to the mechanism by which CD28 and PKCθ contribute to signal transduction in TCR/CD28-engaged T cells. These findings also raise new questions relevant to the mechanism of interaction of CD28 and PKCθ and their specific role in the induction of distinct T cell-mediated immune responses. One obvious question relates to the mechanism by which PKCθ is sequestered away from the IS of activated T_reg_ cells. It would be interesting to determine whether a CD28-Lck-PKCθ tri-partite complex (Kong et al., 2011) occurs in T_reg_ cells, and determine the mechanism that enables PKCθ recruitment away from the T_reg_-APC contact area. A possible explanation for this process was provided by Yokosuka et al. (2010) showing that CTLA-4 competes with CD28 in recruitment to the cSMAC. In addition, it is not known whether PKCθ is involved in a second signal delivery during the costimulation of γδ T cells (Ribot et al., 2011).

Despite the extensive amount of studies on the biology of PKCθ in mouse T cells, very little is known about its regulation and function in human T cells. This is a substantial gap that would need to be filled if PKCθ is destined to fulfill its promise as a clinically relevant drug target (Altman and Kong, 2012). As discussed earlier, the dependence of T cell-mediated deleterious autoimmune/inflammatory responses, including GvHD, on PKCθ, but its dispensable role in beneficial responses (antiviral immunity and GvL response) make it an attractive clinical drug target with potentially advantage over global immunosuppressive drugs such as calcineurin inhibitors (e.g., cyclosporine A), which have pronounced toxic side effects. Indeed, there has been considerable interest among pharmaceutical companies in developing small molecule selective PKCθ catalytic activity inhibitors, and AEB071,the most advanced of these compounds, which inhibits other PKC family members in addition to PKCθ, is currently in early clinical trials (Evenou et al., 2009).

Nevertheless, small molecule inhibitors of protein kinases often have toxic side effects because of their lack of absolute specificity, which reflects the relatively high conservation of catalytic domains within the protein kinase family, and even more so within the PKC family. Furthermore, since catalytic kinase inhibitors in current clinical use are ATP competitors, they need to be used at relatively high and potentially toxic concentrations in order to effectively compete with ATP, whose intracellular concentration is ~1 mM. As a result, there has recently been considerable interest and progress in developing allosteric kinase inhibitors, which bind to sites other than the catalytic site in kinases and, thus, are likely to be much more selective and less toxic (Lamba and Ghosh, 2012). Our recent study (Kong et al., 2011) demonstrates a new potential approach for attenuating PKCθ-dependent functions utilizing allosteric compounds based on the critical PR motif in the V3 domain of PKCθ that will block its Lck-mediated association with CD28 and recruitment to the IS, which is obligatory for its downstream signaling functions. This new approach could serve as a basis for the development of new therapeutic agents that would selectively suppress undesired T cell-mediated inflammation and autoimmunity or prevent graft rejection, while preserving desired immunity, such as antiviral responses.

## Conflict of Interest Statement

The authors declare that the research was conducted in the absence of any com mercial or financial relationships that could be construed as a potential conflict of interest.
